# Supplementary material for: RGS2 drives male aggression in mice via the serotonergic system
Source: Commun Biol. 2019 Oct 11;2:373. doi: 10.1038/s42003-019-0622-0 (PMC6789038; doi:10.1038/s42003-019-0622-0)
Supplement: Supplementary file 1 — Supplementary Information [file 42003_2019_622_MOESM1_ESM.pdf]

Supplementary Information

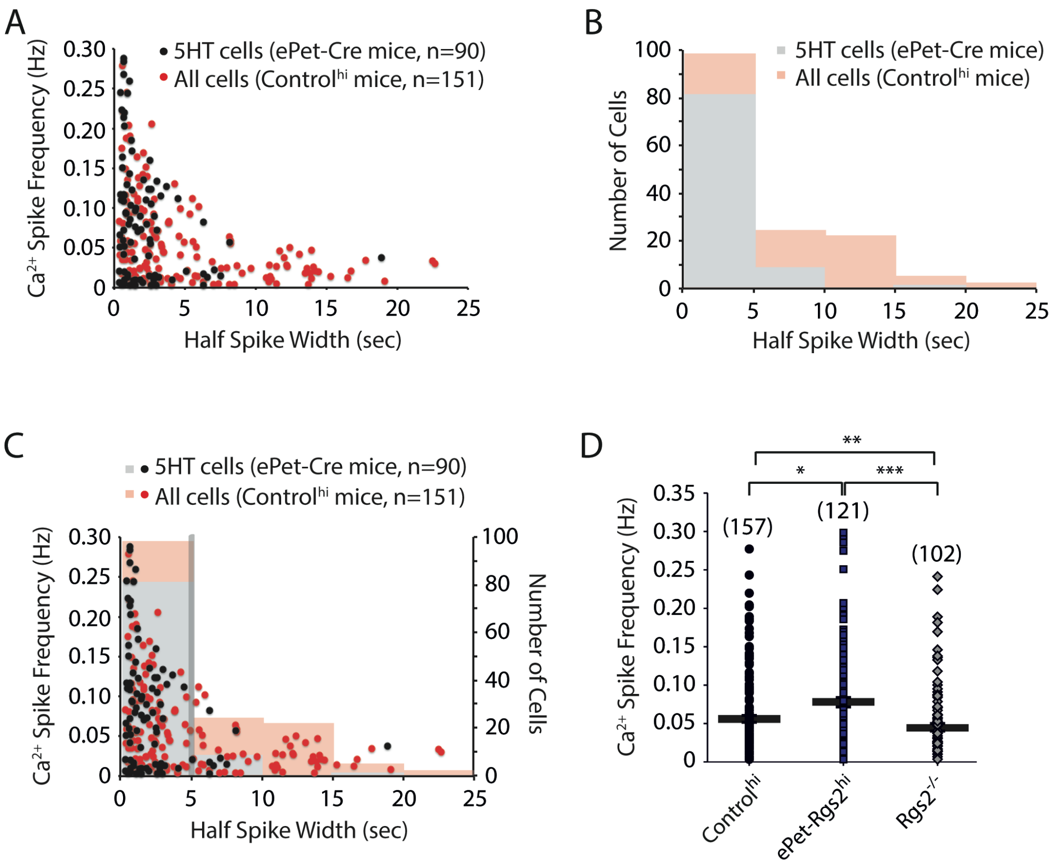

Supplementary Figure 1 Comparison between the Ca^{2+} spike frequency and half spike width between dorsal raphe nuclei (DRN) neurons infected with GCaMP6 from ePet-Cre

and control^{hi} mice. (A) Comparison between the Ca^{2+} spike frequency (Hz) blotted against the half spike width (sec) for DRN neurons infected with an AAV driving the expression of GCaMP6 in all neurons (5HT and non-5HT neurons in the DRN) in control^{hi} mice (red dots) and a floxed version specifically in 5HT neurons in ePet-Cre mice (black dots). **(B)** Histogram of the Ca^{2+} spike frequency between DRN neurons infected with GCaMP6 from ePet-Cre and control^{hi} mice blotted against the half spike width with a bin size of 5 sec. **(C)** Overlay of blot **(A)** and **(B)**. The grey bar shows the half width constrain at 5 sec, which was applied to correct the Ca^{2+} spike frequency measurements for nonspecific 5HT neuron infections. The blots A-C show that in GCaMP6 infected DRN neurons of control^{hi} mice representing 5HT and non-5HT neurons a certain percentage of neurons reveal a broad GCaMP6 signal, which is observed to a much lesser extent in 5HT neurons from ePet-Cre mice. These results suggest that a certain population of non-5HT neurons have a broad GCaMP6 signal. Under the assumption that these cells are non-5HT neurons, we excluded these cells and recalculated the spike frequencies for the remaining cells of DRN neurons infected with GCaMP6, but not 5HT specific AAV expressing GCaMP6 (shown in Fig. 6B). Since 38% of neurons within the DRN/MRN are non-serotonergic neurons [1], we used a half-width constraint of 5 sec (grey bar). Using this constraint 38% of the neurons recorded in control^{hi}, 36% of ePet-RGS2^{hi} and and RGS2$^{-/-}$ mice were excluded. (Note, that for the 5 sec constraint only 10% of 5HT neurons from ePet-Cre mice had a larger half-width than 5). The spike width corrected values are given in Fig. 6B. **(D)** Ca^{2+}spike rates of all analyzed GCaMP6 infected neurons in the DRN between control^{hi}, ePet-RGS2^{hi} and RGS2$^{-/-}$ mice. The number of cells tested/group is indicated in parentheses. Statistical significance was evaluated with ANOVA (*p<0.05, **p<0.01, ***p<0.001).

1. Beck, S. G., Pan, Y. Z., Akanwa, A. C. & Kirby, L. G. Median and dorsal raphe neurons are not electrophysiologically identical. *J Neurophysiol* **91**, 994–1005 (2004).
