# Supplementary material for: RGS2 drives male aggression in mice via the serotonergic system
Source: Commun Biol. 2019 Oct 11;2:373. doi: 10.1038/s42003-019-0622-0 (PMC6789038; doi:10.1038/s42003-019-0622-0)
Supplement: Supplementary file 2 — Description of Additional Supplementary Files [file 42003_2019_622_MOESM2_ESM.pdf]

Description of Additional Supplementary Items

Supplementary Movie 1 Resident intruder test from a control^{hi} male mouse on the 10th day of being exposed to an intruder mouse (black stripe on tail) displaying nonaggressive behavior.

Supplementary Movie 2 Resident intruder test from an ePet-Rgs2^{hi} male mouse (black stripe on tail) on the 10th day of being exposed to an intruder mouse exhibiting aggressive behavior.

Supplementary Movie 3 Tube dominance test of an ePet-Rgs2^{hi} male mouse forcing their control^{hi} littermate out of the tube.

Supplementary Movie 4 Resident intruder test from a Rgs2$^{-/-}$ male mouse on the 10th day of being exposed to an intruder mouse (black stripe on tail) displaying nonaggressive behavior.

Supplementary Movie 5 Resident intruder test from a Rgs2$^{-/-}$/ePet-Rgs2^{hi} male mouse on the 10th day of being exposed to an intruder mouse (black stripe on tail) exhibiting aggressive behavior.

Supplementary Movie 6 Tube dominance test of a Rgs2$^{-/-}$ male mouse willingly exiting the tube after encountering his Rgs2$^{-/-}$/ePet-Rgs2^{hi} male opponent.

Supplementary Movie 7 Tube dominance test of a Rgs2$^{-/-}$/ePet-Rgs2^{hi} male mouse forcing ePet-Rgs2^{hi} male mouse out of the tube.

Supplementary Data An excel file including data for all figures and supplementary figure in article. Additional data is available upon request by the corresponding author.
