# Supplementary material for: RGS2 drives male aggression in mice via the serotonergic system
Source: Commun Biol. 2019 Oct 11;2:373. doi: 10.1038/s42003-019-0622-0 (PMC6789038; doi:10.1038/s42003-019-0622-0)
Supplement: Supplementary file 4 — Reporting Summary [file 42003_2019_622_MOESM4_ESM.pdf]

nature research

Corresponding author(s): Melanie D Mark

Last updated by author(s): 9/3/2019

Reporting Summary

Nature Research wishes to improve the reproducibility of the work that we publish. This form provides structure for consistency and transparency in reporting. For further information on Nature Research policies, see Authors & Referees and the Editorial Policy Checklist.

Statistics

For all statistical analyses, confirm that the following items are present in the figure legend, table legend, main text, or Methods section.

n/a	Confirmed	
☐	☒	The exact sample size (n) for each experimental group/condition, given as a discrete number and unit of measurement
☐	☒	A statement on whether measurements were taken from distinct samples or whether the same sample was measured repeatedly
☐	☒	The statistical test(s) used AND whether they are one- or two-sided<br>*Only common tests should be described solely by name; describe more complex techniques in the Methods section.*
☒	☐	A description of all covariates tested
☐	☒	A description of any assumptions or corrections, such as tests of normality and adjustment for multiple comparisons
☐	☒	A full description of the statistical parameters including central tendency (e.g. means) or other basic estimates (e.g. regression coefficient) AND variation (e.g. standard deviation) or associated estimates of uncertainty (e.g. confidence intervals)
☐	☒	For null hypothesis testing, the test statistic (e.g. F, t, r) with confidence intervals, effect sizes, degrees of freedom and P value noted<br>*Give P values as exact values whenever suitable.*
☒	☐	For Bayesian analysis, information on the choice of priors and Markov chain Monte Carlo settings
☒	☐	For hierarchical and complex designs, identification of the appropriate level for tests and full reporting of outcomes
☒	☐	Estimates of effect sizes (e.g. Cohen's d, Pearson's r), indicating how they were calculated

Our web collection on statistics for biologists contains articles on many of the points above.

Software and code

Policy information about availability of computer code

Data collection	ImageJ (Fiji contributors version); Leica Application Suite (for Leica TCS SP5 confocal laser scanning microscope); Matlab software (vR2014b)
Data analysis	ImageJ (Fiji contributors version); Wavemetrics Igor Pro Software (v6.0);Microsoft Excel (2016); Systat Software Sigmaplot (v12.5); Matlab software (vR2014b)

For manuscripts utilizing custom algorithms or software that are central to the research but not yet described in published literature, software must be made available to editors/reviewers. We strongly encourage code deposition in a community repository (e.g. GitHub). See the Nature Research guidelines for submitting code & software for further information.

Data

Policy information about availability of data

All manuscripts must include a data availability statement. This statement should provide the following information, where applicable:

- Accession codes, unique identifiers, or web links for publicly available datasets
- A list of figures that have associated raw data
- A description of any restrictions on data availability

The authors declare that all the data supporting the findings of this study are available in the manuscript, figures and supplementary information files. All materials and other data supporting this study are readily available from the corresponding author (Melanie.mark@rub.de) upon reasonable request.

Field-specific reporting

Please select the one below that is the best fit for your research. If you are not sure, read the appropriate sections before making your selection.

☒ Life sciences ☐ Behavioural & social sciences ☐ Ecological, evolutionary & environmental sciences

For a reference copy of the document with all sections, see nature.com/documents/nr-reporting-summary-flat.pdf

Life sciences study design

All studies must disclose on these points even when the disclosure is negative.

Sample size	Sample size was estimated with GPower 3.1
Data exclusions	Mice were excluded from a trial when they refused to complete the task and showed in their previous trials ability to complete the task. Cells in Fig 8 were excluded based on their half width spikes to enrich for serotonergic neurons in the analysis as described in detail in the Supplementary Figure 1. The corrected and non corrected data is presented in the Supplementary Figure 1 and Figure 8. No data was excluded otherwise.
Replication	Replication of results were successful
Randomization	Allocation of samples into experimental groups was random
Blinding	Videos from behavior studies were analyzed by 2 different scientists blindly. Investigators were blinded during the experiments.

Reporting for specific materials, systems and methods

We require information from authors about some types of materials, experimental systems and methods used in many studies. Here, indicate whether each material, system or method listed is relevant to your study. If you are not sure if a list item applies to your research, read the appropriate section before selecting a response.

Materials & experimental systems

n/a	Involved in the study
☐	☒ Antibodies
☒	☐ Eukaryotic cell lines
☒	☐ Palaeontology
☐	☒ Animals and other organisms
☒	☐ Human research participants
☒	☐ Clinical data

Methods

n/a	Involved in the study
☒	☐ ChIP-seq
☐	☒ Flow cytometry
☒	☐ MRI-based neuroimaging

Antibodies

Antibodies used	rabbit anti-GFP (Frontiers Institute Co; GFP-Rb-Af2020, RRID : AB_2571573; 1:1000 dilution factor)<br>goat anti-rabbit-A488 (Molecular Probes; A-11008;1:1000 dilution factor)
Validation	rabbit anti-GFP https://w2.frontier-institute.com/<br>https://www.thermofisher.com/antibody/product/Goat-anti-Rabbit-IgG-H-L-Cross-Adsorbed-Secondary-Antibody-Polyclonal/A-11008

Animals and other organisms

Policy information about studies involving animals; ARRIVE guidelines recommended for reporting animal research

Laboratory animals	C57/Bl6 background mice, males, adults, 1-3 months, P0-3; Rgs2-/-, ePet-Rgs2lo, ePet-Rgs2hi, Rgs2-/-/ePet-Rgs2lo, controllo, Rgs2-/-/ePet-Rgs2hi, controlhi, ePet-YFP
Wild animals	NA
Field-collected samples	NA
Ethics oversight	The present study was carried out in accordance with the European Communities Council Directive of 2010 (2010/63/EU) for care of laboratory animals and approved by a local ethics committee (Bezirksamt Arnsberg) and the animal care committee of North Rhine-Westphalia, Germany, based at the LANUV (Landesamt für Umweltschutz, Naturschutz und Verbraucherschutz,

Nordrhein-Westfalen, D-45659 Recklinghausen, Germany). The study was supervised by the animal welfare commission of the Ruhr-University Bochum. All efforts were made to minimize the number of mice used for this study.

Note that full information on the approval of the study protocol must also be provided in the manuscript.

Flow Cytometry

Plots

Confirm that:

☐ The axis labels state the marker and fluorochrome used (e.g. CD4-FITC).

☐ The axis scales are clearly visible. Include numbers along axes only for bottom left plot of group (a 'group' is an analysis of identical markers).

☐ All plots are contour plots with outliers or pseudocolor plots.

☐ A numerical value for number of cells or percentage (with statistics) is provided.

Methodology

Sample preparation	Continental cultures of hippocampal neurons were prepared according to a modified version of published procedures from mouse pups (P0-3). Dissociated serotonergic neurons were previously enriched with FACS (fluorescence activated cell sorting of live cells) from ePet-YFP+ mice where serotonergic neurons are expressing YFP.
Instrument	Becton Dickinson FACS Aria digital cell sorter equipped with an argon laser
Software	BD FACSAria™ software
Cell population abundance	Cells were passed through a 40 um filter and immediately sorted on a Becton Dickinson FACS Aria digital cell sorter equipped with an argon laser providing excitation of 200 mW at 488 nm. YFP fluorescence was detected with the standard FL2 filter set (560 nm dichroic, 585/42 nm bandpass). Sort pressure was 35 psi with an 85 um nozzle tip driven at 39.5 kHz. Maximum event rate was conservatively limited to 5000/s. Forward scatter height versus width gating was used to eliminate aggregates. YFP fluorescent cells were identified by characteristic red versus orange fluorescence and were sorted directly into tubes containing Trizol (Invitrogen) for subsequent RNA extraction or into L15 media for confirmation of sort purity. A typical sort with 4–5 transgenic embryos at E12.5 yielded 45,000 rostral YFP+ and 30,000 caudal YFP+ 5HT neurons. Using this protocol, we collected 200,000 cells for each of the four cell groups to be profiled: R+ (rostral YFP+), R- (rostral YFP-), C+ (caudal YFP+), and C- (caudal YFP-). For more details, please see the reference Wylie, C. J. et al. Distinct transcriptomes define rostral and caudal serotonin neurons. J Neurosci 30, 670–684 (2010).
Gating strategy	A typical sort with 4–5 transgenic embryos at E12.5 yielded 45,000 rostral YFP+ and 30,000 caudal YFP+ 5HT neurons. Using this protocol, we collected 200,000 cells for each of the four cell groups to be profiled: R+ (rostral YFP+), R- (rostral YFP-), C+ (caudal YFP+), and C- (caudal YFP-). For more details, please see the reference Wylie, C. J. et al. Distinct transcriptomes define rostral and caudal serotonin neurons. J Neurosci 30, 670–684 (2010).

☐ Tick this box to confirm that a figure exemplifying the gating strategy is provided in the Supplementary Information.
